# Circulating anti-SARS-CoV-2 nucleocapsid (N)-protein antibodies and anti-SARS-CoV-2 spike (S)-protein antibodies in an African setting: herd immunity, not there yet!

**DOI:** 10.1186/s13104-021-05570-3

**Published:** 2021-04-20

**Authors:** Amandine Mveang Nzoghe, Marielle Leboueny, Eliane Kuissi Kamgaing, Anicet Christel Maloupazoa Siawaya, Eliode Cyrien Bongho, Ofilia Mvoundza Ndjindji, Guy-Stephan Padzys, Bénédicte Ndeboko, Simon Ategbo, Joel Fleury Djoba Siawaya

**Affiliations:** 1 Service Laboratoire, Centre Hospitalier Universitaire (CHU) - Mère-Enfant), Fondation Jeanne EBORI, Libreville, Gabon; 2 Pôle enfant, Service de Néotatologie, CHU- Mère-Enfant Fondation Jeanne EBORI, Libreville, Gabon; 3 Pôle Enfant Service de Pédiatrie, CHU- Mère-Enfant Fondation Jeanne EBORI, Libreville, Gabon; 4 Département de Biologie Cellulaire Et Physiologie, Faculté Des Sciences, Université Des Sciences Et Techniques de Masuku, Franceville, Gabon; 5 Université Des Sciences de La Santé, Libreville, Gabon

**Keywords:** SARS-CoV-2, Antibodies, Infants, Children, Adults

## Abstract

**Objective:**

Herd immunity is achieved when in a population, immune individuals are in a sufficiently large proportion. Neutralizing antibodies specific to SARS-CoV-2 that are produced following infection or vaccination are critical for controlling the spread of COVID-19. The objective of the present work was to investigate the rate of SARS-CoV-2 natural immunization in Gabonese.

**Results:**

One thousand, four hundred and ninety two people were enrolled. The overall prevalence of anti-SARS-CoV-2 antibodies was 36.2%. Moreover, 76.4% of people who developed a humoral response to SARS-CoV-2 produced both anti-SARS-CoV-2 N-protein antibodies and anti-SARS-CoV-2 S-protein antibodies, which correspond to 27.7% of the total population. In infants (0–9 month), children (1–17 years) and adults, the prevalence of anti-SARS-CoV-2 antibodies was relatively the same, between 33 and 37% (any antibody types) and between 25 and 28.6% (neutralizing antibodies). In this African context, one-third (1/3) of the screened population was exposed to SARS-CoV-2 and three-quarter (3/4) of those exposed individuals developed neutralizing antibodies against SARS-CoV-2. This data suggest that herd immunity is not yet to be achieved in Gabon.

## Introduction

The severe acute respiratory syndrome coronavirus 2 (SARS-CoV-2), the causative agent of COVID-19 is spreading around the globe. Many countries, if not all, are now facing this pandemic. Data shows that all continents and countries are not equal, and have in some aspects, their COVID-19 epidemic profiles [1–3]. The main argument for Africa’s resilience has been the younger age of its population. We need to understand why the virus spreads at different speeds and affects populations differently. To understand why the virus affects populations differently, and assess if SARS-CoV-2 herd immunity should be contemplated naturally or as a result of wide-scale vaccination programs [4], there is a need to conduct sero-epidemiological investigations.

In Gabon, more than 200 000 subjects were screened for SARS-CoV-2 infection (by PCR) from the 13th of March- when the first COVID-19 case was reported- to October 2020. The PCR based prevalence of SARS-Cov-2 was 4.4% with a death rate among detected cases of 0.6% (country data). The median age in Gabon is around 20 years old, with more than 60% of the population under 25 years old. It is in that context that an age-stratified sero-epidemiological study was conducted to determine the extent of SARS-CoV-2 exposure in parts of the population. More precisely we determined the prevalence of anti-SARS-CoV-2 nucleocapsid (N)-protein antibodies and anti-SARS-CoV-2 spike (S)-protein antibodies in Gabonese infants, children and adults.

## Main text

### Methods

A prospective study was conducted from July to October 2020 in the setting of the Mother and Child University Hospital (CHUMEJE) in Libreville to establish the prevalence of anti-SARS-CoV-2 antibodies by demographic strata. The approach consisted of testing serum from routine activities.

The detection of anti-SARS-CoV-2 antibodies were done using two tests. The first test was the Elecsys^®^ Anti-SARS-CoV-2 immunoassay (Roche Diagnostics, France). The Roche test detects pre-dominantly IgG, but also IgA and IgM to SARS-CoV-2 N-protein with a sensitivity of 99.5% and a specificity of 99.8%)). The second test was the VIDAS^®^ SARS-COV-2 IgM/IgG test targeting the Spike protein subdomain (S1/RBD) with a manufacturer declared sensitivity of 96.4–100% and a specificity of 100% (Biomerieux, France). Both tests’ cutoff index (COI) was one. Assays were conducted following the manufacturers' strict instructions.

The hospital board approved the study, and samples from consenting participants were selected for analysis. Consent was informed and obtained in a written or verbal format (both formats are allowed) based on participant preferences.

### Results

One thousand, four hundred and ninety five people were included in the study: 110 infants aged 9 months and below (7.4%), 141 children between the age of 1 and 5 years old (9.4%), 143 children aged between 6 and 17 years old (9.6%), 993 women aged between 18 and 85 years old (66.5%) and 108 men aged between 18 and 78 years old (7.2%).

The overall prevalence of anti-SARS-CoV-2 antibodies was 36.2%. 27.7% of people developed both anti-SARS-CoV-2 nucleocapsid (N)-protein antibodies and anti-SARS-CoV-2 spike (S)-protein antibodies. Anti-SARS-CoV-2 antibodies prevalence in infants (0–9 months), children (1–17 years), and adults (men and women) were relatively the same, ranging between 33 and 37% (all antibody types) and between 25 and 28.6% (both anti-N-protein and anti-S-protein antibodies). Our analysis also showed that 76.4% of anti-SARS-CoV-2 antibody-positive subjects had both anti-SARS-CoV-2 N-protein antibodies and anti-SARS-CoV-2 S-protein antibodies. 22.4% of subjects positive for anti-SARS-CoV-2 antibody-positive had only anti-SARS-CoV-2 N-protein antibodies and 1.2% of subjects positive for anti-SARS-CoV-2 antibody-positive had only anti-SARS-CoV-2 S-protein antibodies.

The Chi-square test of significance showed no differences in immunization status by gender or by age groups. Table 1 shows the prevalence of anti-SARS-CoV-2 antibodies in the studied populations.

**Table 1:** Age and gender stratified prevalence of anti-SARS-CoV-2 antibodies

	Age	N	Positive for SARS-CoV-2 antibodies – any test N (%)	Positive for both anti-N-protein and anti-S-protein antibodies N (%)	Crude prevalence (Any type of antibody) (%)	Prevalence of both anti-N-protein and anti-S-protein antibodies (%)
Infants (N = 110)	0–3 months	68	25 (37)	19 (28)	36	27.2
	4–9 months	42	15 (36)	11 (26.2)		
Children (N = 284)	1–5 years old	141	44 (31)	34 (24.1)	33	25
	6–17 years old	143	49 (34)	37 (26)		
Women (N = 993)	18–44 years old	845	324 (38.3)	247 (29.2)	37	28.5
	45–85 years old	148	47 (32)	36 (24.3)		
Men (N = 105)	18–44 years old	63	21 (33)	16 (25.4)	37	28.6
	45–78 years old	42	18 (43)	14 (33.3)		

N = number of participants; % percentage of participants (prevalence)

### Discussion

Similar to the study by Anand et al., our study used blood collected as part of routine medical care, limiting selection bias [5]. Libreville is the epi-centre of SARS-CoV-2 infection in Gabon, concentrating 72% of cases (country data). In our analysis of the prevalence of SARS-CoV-2 antibodies from patients attending the Libreville mother and child university hospital laboratory services showed that evidence of SARS-CoV-2 exposure in 36.2% of tested people. The prevalence of SARS-CoV-2 antibodies was comparable in all groups (children and adults). 27.7% of the population developed both anti-SARS-CoV-2 N-protein antibodies and anti-SARS-CoV-2 S-protein antibodies. Data revealed that 76.4% of people who developed a humoral response to SARS-CoV-2 produced both anti-SARS-CoV-2 N-protein antibodies and anti-SARS-CoV-2 S-protein antibodies. This finding is interesting as it suggests that at least 76.4% of people naturally exposed to SARS-CoV-2 antigens most probably develop protective immunity.

Currently, there is limited data on mother-to-child passive transfer of anti-SARS-CoV-2 antibodies. With 28% of newborn infants (aged 0 to 3 months) showing evidence of antibodies against both SARS-CoV-2 N- and S-protein, our study brings further evidence of mother-to-child transfer of protective immunity.

Overall, with only 36.2% of people presenting evidence of circulating anti-SARS-CoV-2 antibodies and 27.7% had developed a neutralizing immunity, it is safe to presume that Gabon is not near reaching herd immunity. Although there is a very limited number of reports on anti-SARS-CoV-2 antibodies seroprevalence in Africa, we believe that like Gabon, a great number of African countries are far from naturally acquired herd immunity [6, 7]. In Kenya, the crude seroprevalence of anti-SARS-CoV-2 antibody was 4.3% [7]. The prevalence of anti-SARS-CoV-2 antibody observed in South-African ranged from 32 to 63% [6]. Worldwide the pooled seroprevalence of anti-SARS-CoV-2 antibodies was estimated at 3.6% (with a maximum of 22%) [8]. These indicators show a very disparate SARS-CoV-2 exposure across communities and suggest that naturally acquired herd immunity is yet to be achieved worldwide. African immunization through vaccine remains therefore relevant.

The initiative COVAX works to guaranty access to COVID-19 vaccines in all parts of the world, the provision of vaccines for Africa is very limited [9]. We would argue that a great number of African countries will be forced to implement a kind of infection-based herd immunity policy (i.e., letting the low-risk people be naturally exposed to the virus while vaccinating people at higher risk) [10].

### Conclusion

The study showed that one-third (1/3) of the screened population was exposed to SARS-CoV-2 and three-quarter (3/4) of those exposed individuals developed neutralizing antibodies against SARS-CoV-2. The relatively low rate of immunization in this study suggests that we are yet to reach herd immunity in this population**.**

## Limitation

The principal limitation of the study is the small number of subjects in the age-stratified groups of infants and men.

## Data Availability

The dataset on which this paper is based on (documentation, raw data file, and methods) used to support this study is available upon request from Prof Joel Fleury DJOBA SIAWAYA (joel.djoba@gmail.com).

## Abbreviations

CHU: Centre Hospitalier Universitaire
COVID-19: Coronavirus disease 2019
COVAX: COVID-19 vaccine access
IgG: Immunoglobulin
PCR: Polymerase chain reaction
SARS-CoV-2: Severe acute respiratory syndrome coronavirus 2
